# 3D Characterization of Acetabular Deficiency in Children with Developmental Dysplasia of the Hip

**DOI:** 10.1007/s43465-021-00458-7

**Published:** 2021-07-23

**Authors:** Raghav Badrinath, Megan E. Jeffords, James D. Bomar, S. Imraan Ahmed, Andrew T. Pennock, Vidyadhar V. Upasani

**Affiliations:** 1 Department of Orthopedics and Rehabilitation, University of California - San Diego, 200 W. Arbor Drive, MC 8894, San Diego, CA 92103 USA; 2 Orthopedics and Scoliosis, Rady Children’s Hospital San Diego, 3020 Children’s Way, MC 5062, San Diego, CA 92123 USA; 3 Pediatric Orthopedics, 9300 Dewitt Loop, Fort Belvoir, VA 22060 USA

**Keywords:** Acetabular dysplasia, Computed tomography, Quantitative analysis, Developmental dysplasia of the hip

## Abstract

**Background:**

The purpose of this study is to determine if a quantitative method can be used to identify differences in 3D morphology between normal and developmentally dysplastic hips and to identify specific areas of undercoverage in children with DDH compared to age- and sex-matched controls.

**Methods:**

Subjects were included if they were typically developing children with no other underlying conditions affecting their musculoskeletal system and had an available pelvic CT scan (67 hips). Custom software was used to measure standard variables defining acetabular morphology (version, tilt, surface area). Acetabuli were divided into equal octants; coverage angles were measured for each octant of interest. Variables were compared with age- and sex-matched controls (128 hips) using analysis of variance or the Mann–Whitney test.

**Results:**

Hips with DDH were more anteverted compared to normal hips (DDH: 22.6˚, Control: 16.4˚, *p* < 0.001). The surface area was similar between groups. 28% of hips had a global deficiency, 24% were anteriorly deficient, 19% were laterally deficient, 10% were anteverted (under covered anteriorly and over covered posteriorly), 3% were posteriorly deficient, and 15% of hips had borderline undercoverage. None of the hips in this cohort were found to be retroverted.

**Conclusions:**

This is the first study to quantify the 3D acetabular deficiency in children with DDH compared to age- and sex-matched controls. We found wide variability in coverage patterns among dysplastic hips. It is imperative to define the specific acetabular deficiency for each individual patient prior to surgical correction.

**Level of evidence:**

III – Case–control study.

## Introduction

Developmental dysplasia of the hip (DDH) is the most common hip disorder of childhood. DDH has long been recognized as a mechanical cause of early osteoarthritis of the hip [1–6]. It is thought that the decreased weight-bearing surface area in dysplastic hips results in increased contact forces and subsequent accelerated osteoarthritis. In many cases, dysplasia improves with bracing, allowing reciprocal forces between a well-located femoral head and the acetabulum to correct the dysplastic hip. However, in a small proportion of children, residual dysplasia persists despite bracing, necessitating surgical intervention to correct the mechanical imbalance and restore acetabular shape and orientation [5, 7].

Treatment strategies aim to ensure a stable and reduced hip within the acetabulum with appropriate depth and weight-bearing coverage area. Ganz et al. report that the primary aim of hip preserving surgical treatment is to improve coverage and congruence of the femoral head in the acetabulum, thereby allowing for more favorable mechanics [8]. Acetabular anatomy is complex, and multiple pelvic and acetabular deformities can coexist. The advent of computerized tomography (CT), and more recently, 3-dimensional CT, have vastly improved our ability to quantify this deformity [9–12].

The importance of precisely understanding the pathoanatomy of the disease prior to surgical treatment has long been recognized [9, 10, 13–17]. Reorientation acetabuloplasties typically aim to improve on this deficiency by increasing anterosuperior coverage. However, given the wide variation and complexity between individuals, performing a standard acetabuloplasty could potentially inadequately address the underlying dysplasia, or produce additional deformity. Indeed, studies have demonstrated iatrogenic retroversion and excessive external rotation following pelvic osteotomies [13, 15, 17]. Authors have consequently attempted to delineate the anatomy of the acetabulum in DDH using cross-sectional imaging. However, these have noted drawbacks. They have primarily been performed in small series of patients, use qualitative methodology, or lack comparison to a control group to define abnormal. We, therefore, aimed to use quantitative measurements using 3D CT scans to define specific morphological characteristics of acetabuli in patients with DDH. Additionally, we aim to identify the primary regions of undercoverage in patients with DDH compared to age- and sex-matched controls. We hypothesize that children will have specific areas of acetabular deficiency that should be recognized and addressed during the surgical treatment.

## Methods

Following internal review board approval, we identified all children with DDH treated at a single institution between 2005 and 2016. Subjects were included if they were over 8 years of age, were typically developing children with no other underlying conditions affecting their musculoskeletal system, and had an available pelvic CT scan. We retrospectively reviewed 40 children with a diagnosis of DDH (31 female, 9 male) with a lateral center edge angle (LCEA) < 25° (measured on plain radiographs) who had undergone pre-operative fine cut CT scans of the pelvis. Five hips were excluded because they had a prior operative intervention on the acetabulum, or they had evidence of inflammatory hip disease documented in the chart. Eight hips had to be further excluded as poor 3D resolution precluded accurate analysis within the custom software. Sixty-seven dysplastic hips were included and analyzed. Of these 67 hips, 50 (75%) had no prior treatment for DDH, nine hips (13%) had a previous open reduction, five hips (7%) had previous brace treatment only, and the remaining three hips (4%) had a prior surgical reduction at an outside institution, it is unknown if this prior reduction maneuver was performed open or closed.

CT scans were imported into 3D image processing software (Mimics, Materialize NV, Leuven, Belgium) as described by Upasani et al. [18]. The software was used, in conjunction with manual input, to selectively identify bony anatomy and create surface 3D models using image segmentation. Pelvis 3D models were exported in stereolithography (STL) format and imported into custom software written in MATLAB (Mathworks, Natick, MA).

The pelvic position was standardized in the anterior pelvic plane utilizing the anterior superior iliac spine (ASIS) and pubic tubercle. The superior portion of the right and left iliac spines were aligned in the coronal plane and the right and left ASIS were aligned in the axial plane. Acetabulum surfaces on the pelvic models were automatically identified and a best-fit sphere utilizing least-squares regression was used to approximate the acetabulum center of rotation. This allowed independent acetabulum analysis. Surface boundaries were next traced using the custom MATLAB program.

**Fig. 1:**
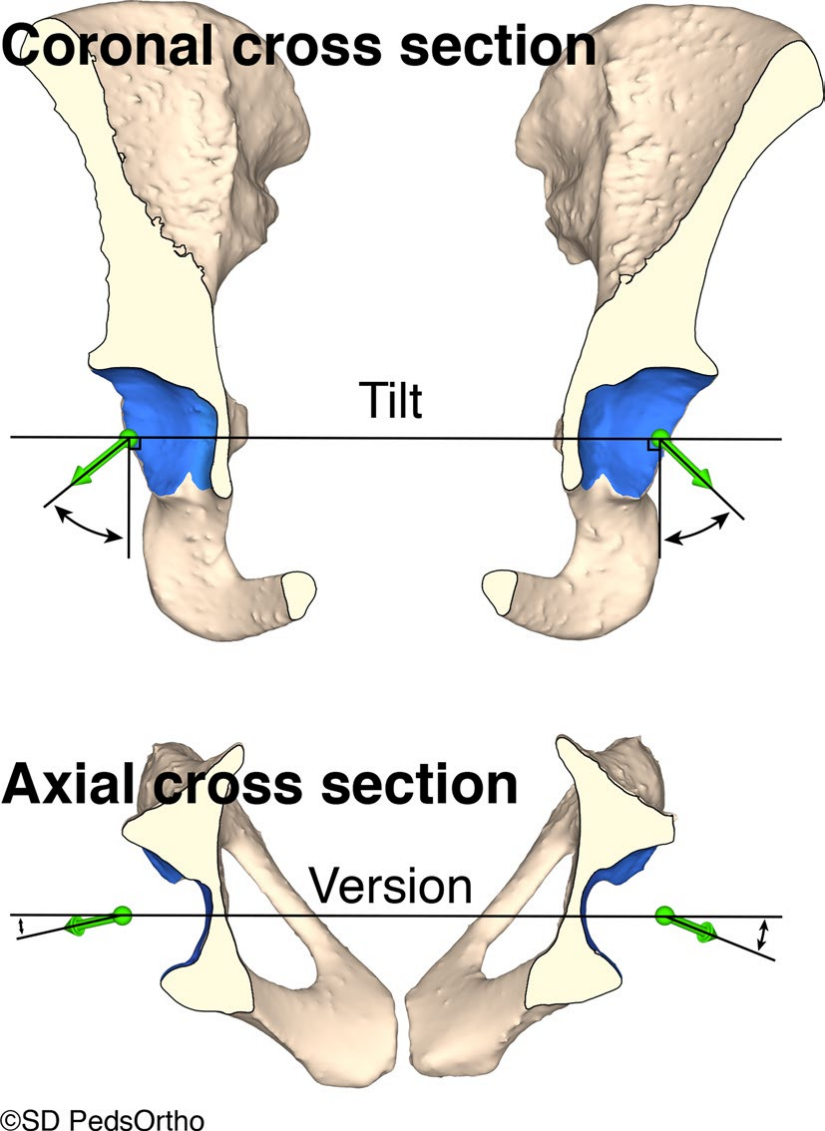
Illustration of how tilt and version were calculated. (top) Tilt is calculated in the coronal plane using the direction vector and a line perpendicular to the line connecting the center of the best-fit spheres of each hip. (bottom) Version is calculated in the axial plane using the direction vector and the line connecting the center of the best-fit spheres of each hip

The surface area was determined after surface boundary tracing. The triangular surface normal vectors were integrated over the entirety of the mapped acetabulum to calculate the acetabular direction vector. Acetabular tilt was evaluated in the coronal plane. First, a line connecting the center of the best-fit spheres of each hip was made. A second line, perpendicular to the first was also made. The angle between this perpendicular line and the direction vector is tilt (Fig. 1). The acetabular version was evaluated in the axial plane. The same horizontal line connecting the center of the best-fit spheres that was used to evaluate tilt was used to evaluate version. Version is the angle between this horizontal line and the direction vector in the axial plane (Fig. 1).

**Fig. 2:**
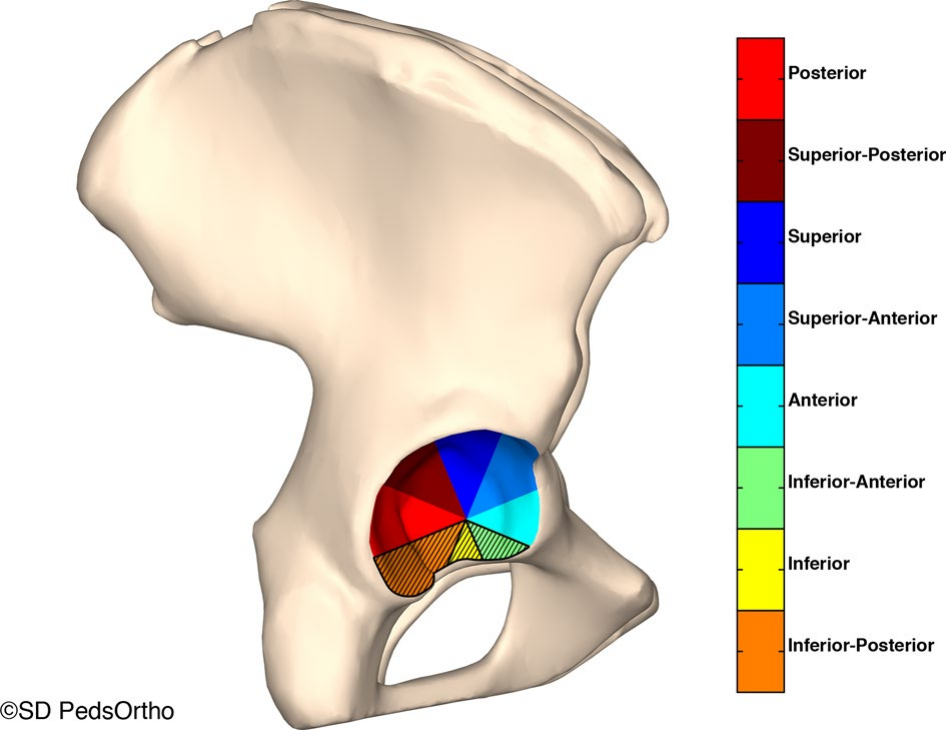
Along a radial axis parallel with the horizontal plane the acetabulum was divided into 8 radial sectors. The sectors were named superior (S), superior anterior (SA), anterior (A), inferior anterior (IA), inferior (I), inferior posterior (IP), posterior (P), and superior posterior (SP). The inferior sectors are removed from the analysis as they play no role in femoral head coverage

**Fig. 3:**
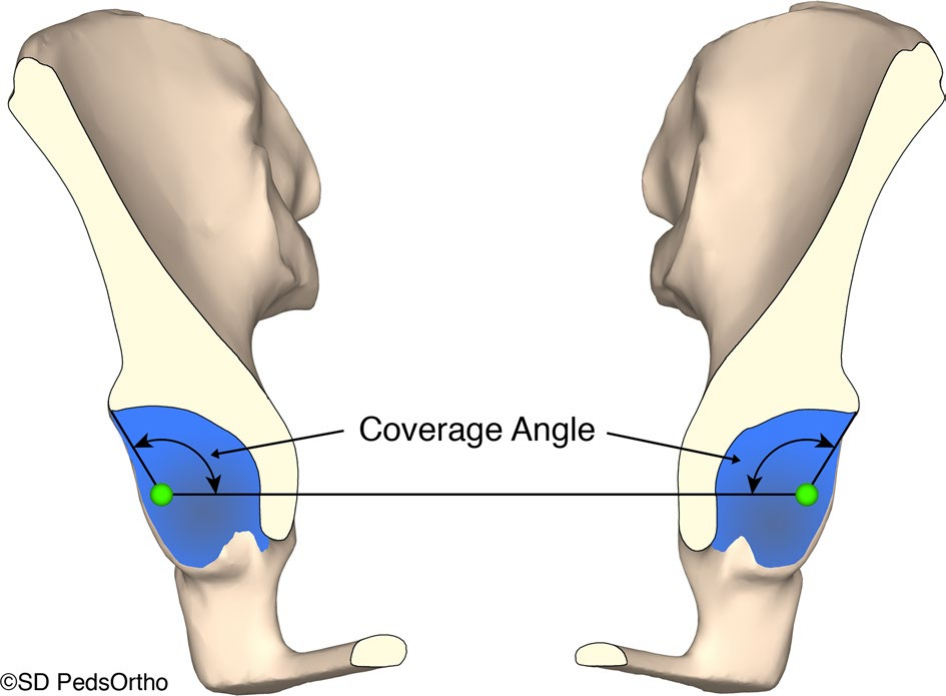
**a**–**d** Coverage angles were calculated by measuring the angle between the line connecting the center of the best fit sphere of each hip and the edge of the acetabulum. This measurement was performed in a continuous radial fashion rotating along the axis of the line connecting the center of the two best-fit spheres. The measurement for each sector was an average of the radial measurements that were taken in each specific sector

Along a radial axis parallel with the horizontal plane, the acetabulum was divided into 8 radial sectors. The sectors were named superior (S), superior anterior (SA), anterior (A), inferior anterior (IA), inferior (I), inferior posterior (IP), posterior (P), and superior posterior (SP). The inferior octants were dropped from this analysis because they do not contribute to femoral head coverage. The 12 and 6 o’clock positions bisect the superior and inferior slices, respectively (Fig. 2). Coverage angles were next measured and defined as the angle between the left–right axis and the line connecting the acetabulum center with the edge of the acetabulum (Fig. 3). Mean coverage angle was taken for sectors P, SP, S, SA, and A.

Previously published control values generated with this identical technique were utilized [19]. These control values were obtained from CT pelvis scans obtained in the emergency department (ED) for non-orthopedic conditions as part of the ED’s standard workup protocol. Prior to analysis of the control cohort, retrospective IRB approval was obtained and a chart review was performed to confirm that none of the subjects had a history of, or current orthopedic conditions affecting the hip and that all subjects were typically developing individuals. In all, 128 hips formed part of the control group data set. Patients in the control group had a CT scan for a variety of reasons, the four most common were appendicitis (27%), abdominal pain (17%), gastroenteritis (13%), and constipation (6%).

Statistical analysis was conducted using SPSS (Version 25.0; SPSS, Inc, Chicago, IL, USA). Each hip was referenced to age- and sex-matched controls. The unit of analysis was the hip. Statistical significance was set at a *p* < 0.05. No a priori power analysis was performed. All data cohorts were tested for normality. Mean differences in normal data were evaluated using one-way ANOVA. Non-normal data were evaluated using the Mann–Whitney test. The intraclass correlation coefficient (ICC) was calculated between CE angles measured on plain radiographs and degree of lateral coverage measured using our 3D software to evaluate the correlation of traditional CE angle measures to 3-dimensional data. Coverage values for each radial sector in the dysplastic hips were compared to age and sex-matched controls and identified as under- or over-covered if the z-score was less than or equal to ± 2.0. This was used to categorize hips based on region of acetabular pathology as follows: Anterior, lateral (superior), posterior, global, and anteverted. When classifying each hip into a region of undercoverage, preference was given to the anterior and posterior sectors over the superior sector. For example, if a hip was deficient in the anterior, anterior–superior, and superior sectors, the hip was classified as anteriorly deficient. Lateral undercoverage was classified as a superior deficiency, or a superior deficiency and an adjacent anterior–superior, or posterior–superior deficiency. Globally deficient hips were defined as hips with a minimum of two deficient sectors, occurring a minimum of two sectors apart. Anteverted hips were defined as being over-covered posteriorly, and under-covered anteriorly.

## Results

Sixty-seven dysplastic hips were included in the final data set. Children ranged from eight years of age to 17 years of age, with a median age of 13.5 years (mean: 13.6 ± 2.5 years). There were more female than male hips in the dysplastic group (50 F, 17 M). Table 1 demonstrates the demographics of the two studied groups.

**Table 1:** Cohort demographics

		Dysplastic hips	Control hips
		(*n* = 67)	(*n* = 128)
Sex (*p* = 0.858)	Male	17 (25%)	34 (27%)
	Female	50 (75%)	94 (73%)
Laterality (*p* = 0.921)	Left	34 (51%)	64 (50%)
	Right	33 (49%)	64 (50%)
Age (years)(*p* = 0.953)	Mean	13.6 ± 2.5	13.6 ± 2.6
	Range	8.8 to 17.9	8.4 to 17.9

The mean LCEA for the dysplastic hips was 16.7 ± 7.7° (range: – 9 to 24°). ICC between manual LCEA measurements on plain radiographs and automated lateral coverage angle measures on 3D data was 0.703 (95% confidence interval 0.557 to 0.806, *p* < 0.001), demonstrating a good correlation. Dysplastic hips were found to have increased version and tilt compared to the control group (*p* < 0.001) (Table 2).

**Table 2:** Version, tilt, and surface area

		Dysplastic hips	Control hips
		(*n* = 67)	(*n* = 128)
Version (*p* < 0.001)	Mean ± std. dev	22.6 ± 7.1°	16.4 ± 7.0°
	Range	6.5 to 43.7°	–0.2 to 33.6°
Tilt (*p* < 0.001)	Mean ± std. dev	54.6 ± 3.5°	51.0 ± 4.6°
	Range	47.4 to 63.9°	41.5 to 65.1°
Surface area (*p* = 0.166)	Mean ± std. dev	29.8 ± 5.0 cm^2^	30.6 ± 4.8 cm^2^
	Range	16.3 to 44.2 cm^2^	21.6 to 48.0 cm^2^

Twenty-eight percent (19/67) of dysplastic hips were found to be globally deficient. Anterior deficiency was noted in 24% (16/67) of hips, 19% (13/67) were observed to have a lateral deficiency, 10% (7/67) of dysplastic hips were found to be anteverted (posterior overcoverage and anterior under coverage), two dysplastic hips (3%) were found to be under covered posteriorly. None of the hips in our cohort were found to be retroverted (posterior under coverage and anterior overcoverage). Ten hips in the dysplastic group (15%) were found to have borderline dysplasia defined as a z-score between – 1 and – 2. The mean LCEA of these 10 hips was 21.3 ± 2.5° (range: 17 to 24°).

## Discussion

DDH has long been known to be a disease of altered mechanics. Wiberg, in his seminal 1939 monograph, posited that decreased coverage would result in increased joint reactive forces, and result in accelerated cartilage wear and osteoarthritis [6]. Using standard pelvic radiographs, Wiberg introduced the LCEA angle as a measure of lateral coverage, discovering that all patients in his cohort with an LCEA angle less than 20° developed early radiographic arthritis. Based on his initial studies, an LCEA angle of 20° to 25° continues to be used as representative of a dysplastic hip.

However, multiple studies have demonstrated problems with the reliability of the LCEA angle as a single measure of dysplasia [20–22]. In particular, there has been an increased appreciation of the 3-dimensional complexity of the acetabulum, with the recognition that a single measure of coverage cannot adequately describe the precise acetabular morphology of a dysplastic hip [19].

With the advent of surgical techniques to address the deficient acetabulum, this has significant clinical consequences [13, 15, 17]. Tannast et al., for example, described iatrogenic retroversion following pelvic osteotomies causing worse patient outcomes requiring revision pelvic osteotomies [17]. Recognizing this, other studies have attempted to use 3D CT scans to evaluate the dysplastic acetabulum.

Roach et al. used 3D CT scans to assess the congruence and coverage of the acetabulum and the femoral head using best-fit spheres in 14 patients with DDH, suggesting a new method for quantitative preoperative planning [11]. Ganz et al. similarly reported on their technique of preoperative planning using CT scans in a series of 21 patients [9]. The largest series was by Kim et al., who analyzed CT scans from 70 hips and categorized them based on the region of primary deficiency by qualitatively looking at the shape of the acetabulum. They noted primarily midsuperior deficiency in 38% of the hips, followed by an anterosuperior deficiency in 29% [23]. None of the hips were noted to be primarily deficient posteriorly. A follow-up study examining 41 hips in 24 patients with dysplasia secondary to neuromuscular disease, in contrast, demonstrated primarily posterior deficiency in 37% of the hips studied [23].

Another paper by the same group, using similar methods, demonstrated statistically similar anteversion between dysplastic hips and controls, although with a wide range of values in both groups [24]. In contrast, our study noted that 13% of dysplastic hips were anteverted or posteriorly over covered compared to controls, however, we also observed a wide range of values in both groups. This was corroborated in a recent study fitting acetabular prosthesis to dysplastic acetabuli to determine orientation [25].

**Fig. 4:**
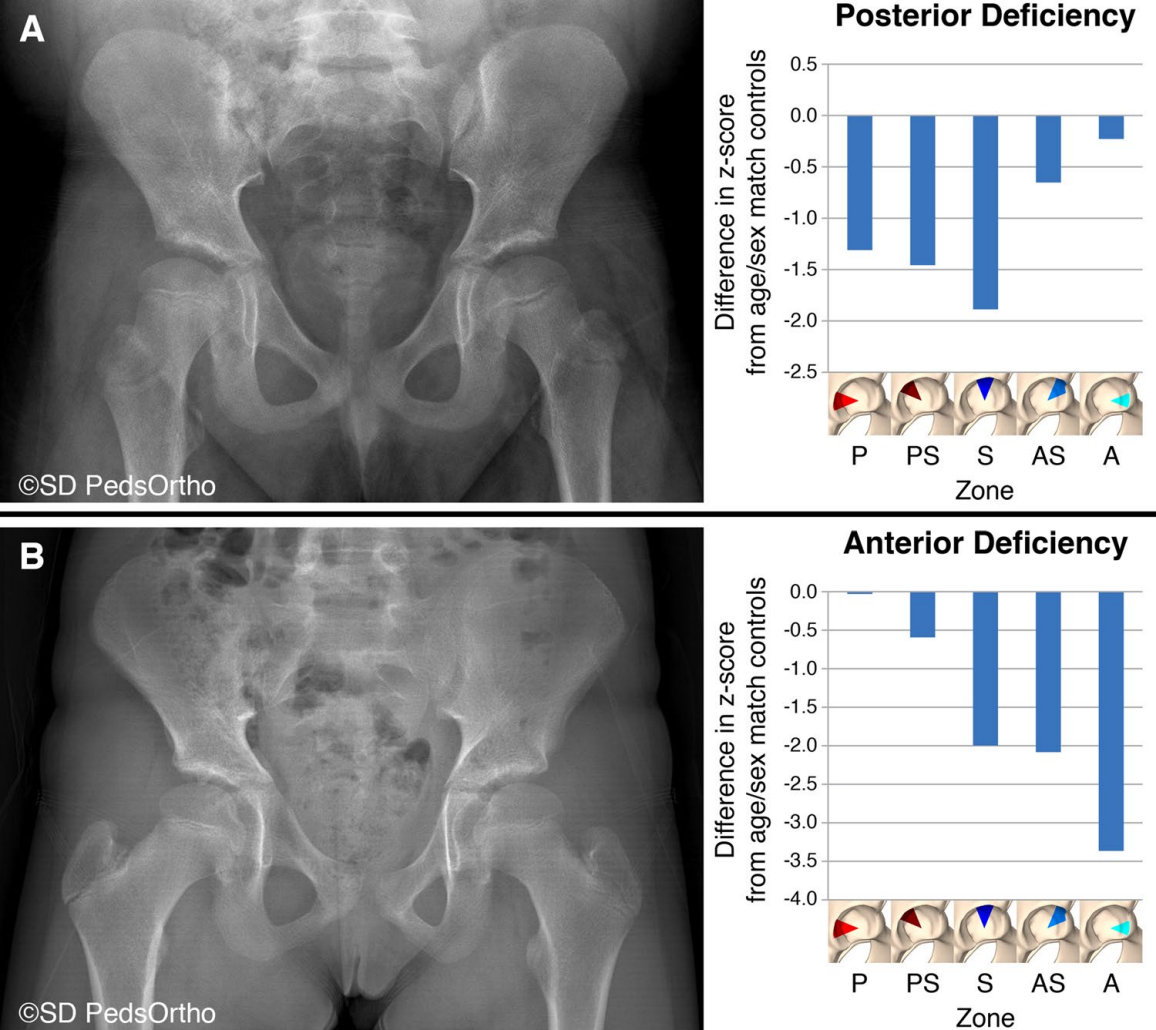
**a**, **b** Radiographs demonstrating two hips with accompanying z-scores by region of undercoverage. The hip in **A** demonstrates primarily posterior undercoverage in the left hip of a 16-year-old. The hip in **B**, on the other hand, demonstrates primarily anterior deficiency in the left hip of a 9-year-old

Our results demonstrate that some hips with DDH are primarily deficient posteriorly. This is similar to the findings of Millis et al. [10] It appears that posterior deficiency is not simply a morphological feature of neuromuscular dysplasia. This has profound implications to preoperative planning for pelvic osteotomies. Attempting to perform a standard pelvic osteotomy to increase anterior coverage would result in worsening of the posterior deficiency, possibly with the development of anterior impingement. Figure 4 demonstrates two hips with accompanying z-scores by region of undercoverage. The hip in Fig. 4A demonstrates primarily posterior undercoverage in the left hip of a 16-year-old. The hip in Fig. 4B, on the other hand, demonstrates primarily anterior deficiency in the left hip of a 9-year-old.

One limitation of our study methodology is that the software used is only able to identify bony morphology, and is inaccurate in cases with significant differences in bony and chondral anatomy. As a consequence, we had to exclude patients below the age of 8 years due to inadequate osseous definition. Additionally, although our data set included a large number of hips in both groups, some age and sex subgroups have small numbers of hips and will need to be expanded in the future. Additionally, this measurement technique can only be performed on a CT scan and cannot be used with other diagnostic exams that require less, or no radiation, such as ultrasound, MRI, or plain radiographs. Finally, our methodology relies on fitting a best-fit sphere to describe the morphology of the acetabulum, which may be insufficient in cases of significant deformity due to disease.

However, despite the limitations, we demonstrate a method to measure radial coverage angles in the dysplastic hip using 3-dimensional reformats of CT scans, with clinical implications. To our knowledge, this represents the largest study quantitatively describing the 3-dimensional morphology of the acetabulum in hips with DDH. Our findings demonstrate wide variance in acetabular morphology in DDH. Consequently, careful recognition of the 3-dimensional deformity in dysplastic hips is important for appropriate pre-operative planning for pelvic osteotomies.
